# Rapid Evaluations of Telehealth Strategies to Address Hypertension: A Mixed-Methods Exploration at Two US Health Systems During the COVID-19 Pandemic

**DOI:** 10.5888/pcd19.220219

**Published:** 2022-12-08

**Authors:** Meera Sreedhara, Kara Suvada, Myles Bostic, Amber Scott, Ethan Blum, Julia Jordan, Kincaid Lowe Beasley

**Affiliations:** 1 Division for Heart Disease and Stroke Prevention, Centers for Disease Control and Prevention, Atlanta, Georgia; 2 Oak Ridge Institute for Science and Education, Oak Ridge, Tennessee; 3 Department of Epidemiology, Emory Rollins School of Public Health, Atlanta, Georgia

## Abstract

Telehealth is a promising intervention for hypertension management and control and was rapidly adopted by health systems to ensure continuity of care during the COVID-19 pandemic. Rapid evaluations of telehealth strategies at 2 US health systems explored how telehealth affected health care access and blood pressure outcomes among populations disproportionately affected by hypertension. Both health systems implemented telehealth strategies to maintain continuity of health care services during the COVID-19 pandemic. The evaluations used a mixed-method approach; qualitative interviews were conducted with key staff, and quantitative analyses were performed on patient electronic health record data. Both health systems exhibited similar trends in telehealth use, which allowed for continued access to health care for some patients but hindered other patients who had limited access to the internet or the equipment needed. Telehealth provides opportunities for blood pressure control and management. Further evaluation is needed to understand the role of broadband internet access as a social determinant of health and its impact on equitable patient access to health care.

**Summary**

**What is already known on this topic?**

Telehealth is a promising intervention for blood pressure control and management. The COVID-19 pandemic accelerated health care systems’ implementation and use of telehealth for continuity of care.

**What is added by this report?**

Rapid evaluations documented telehealth use among patients diagnosed with hypertension in 2 US health care systems during the COVID-19 pandemic. Telehealth offered consistent health care access and opportunities to monitor blood pressure outcomes for some patients. Health inequities may be exacerbated among patients with barriers to telehealth and blood pressure measurement.

**What are the implications for public health practice?**

Telehealth provides opportunities for blood pressure control and management, but the role of telehealth on equitable patient access to health care requires further evaluation.

## Introduction

More than half of US adults who have hypertension have uncontrolled high blood pressure, which increases the risk of cardiovascular disease and stroke (1). Disparities in hypertension and blood pressure control persist, in part, because of structural and systemic inequities (2). The effect of the COVID-19 pandemic on hypertension control is unclear because only a small amount of evidence exists, which is conflicting and did not evaluate the impact among populations at highest risk for hypertension (3–5). 

Telehealth, the use of electronic and telecommunication technologies for health care delivery and education, is recommended for blood pressure control (6). Although the COVID-19 pandemic limited the delivery of office-based primary care visits and blood pressure assessments (7), less stringent federal and state regulations led to the broad expansion of telehealth, including primary care (7,8).

Studies conducted during the COVID-19 pandemic reported changes in telehealth use and subsequent inequities in the general population (8–10). Limited research described the use of telehealth for hypertension control in a primary care setting (3,5). As of March 2022, 20.5% of US adults reported having had a recent telehealth appointment (9), with use during the pandemic varying by such factors as race, ethnicity, age, insurance status, income, language, and urbanicity (8,10). Despite the potential to improve access to health care for people lacking transportation or living in rural areas (11), it is unclear how telehealth has affected access to chronic disease management services during the pandemic (3,5). As national organizations call for the equitable expansion of telehealth (11), practice-based evidence describing the role of telehealth in supporting patients with a diagnosis of hypertension is needed for health care systems to successfully adapt to the shifting landscape of health care delivery.

## Purpose and Objectives

To quickly generate practice-based evidence, we conducted rapid evaluations of 2 US health care systems’ use of telehealth strategies to address hypertension during the COVID-19 pandemic. This study reports on a subset of evaluation findings, which aimed to 1) examine telehealth use among patients with hypertension, with a focus on populations who experience barriers to care and 2) explore the effect of telehealth on blood pressure outcomes.

## Intervention Approach

We evaluated how 2 US health care systems (ARcare and Terros Health) provided telehealth services for patients with hypertension in a primary care setting during the COVID-19 pandemic. Both systems used team-based care and self-measured blood pressure monitoring approaches for hypertension management and control. Telehealth was delivered at both sites via telephone-only and videoconferencing modalities as part of comprehensive primary care and chronic disease management services.

ARcare is a federally qualified health center (FQHC) that comprises 48 clinics that serve 17 counties in Arkansas, Kentucky, and Mississippi. We derived data for this evaluation from ARcare’s headquarters in Augusta, Arkansas. ARcare serves patients who are medically underserved, have low incomes, experience homelessness, and live in rural communities. Telehealth services began in 2018 and were scaled up in March 2020 because of the COVID-19 pandemic. Telehealth is primarily delivered clinic to clinic, where patients visit a clinic outfitted with technology to connect with an off-site health care provider, which allows for collection of data on vital signs and laboratory tests. Telehealth is also delivered at satellite clinics (eg, schools) or to patients at home.

Terros Health (hereinafter, Terros) serves a diverse population at 13 locations in Arizona, including 4 FQHCs. Patients experience health disparities caused by food insecurity, low income, and homelessness, and most patients are uninsured or receive Medicaid. Terros began providing telehealth in March 2020 because of the COVID-19 pandemic. Most patients engage in telehealth from their home. Health care providers can review blood pressure readings if patients have a blood pressure monitor.

## Methods

We conducted a systematic screening and evaluability assessment in 2020 to select health care systems to participate in rapid evaluations (12). Centers for Disease Control and Prevention institutional review board approval was not required for this evaluation project. This analysis reports findings from the mixed-methods outcome evaluation. We triangulated qualitative and quantitative findings to comprehensively describe telehealth strategies, reach to patients, and blood pressure outcomes.

### Qualitative data sources and analysis

We conducted semistructured interviews at each site in June 2021. Two interviewers and a notetaker attended each interview. Informed consent was obtained. A professional service transcribed recorded interviews verbatim.

We tailored interview guides by staff type and in alignment with the evaluation questions to understand the telehealth strategies, reach to patients, and perceived impact on health. We interviewed health system leaders, practice managers, telehealth implementation leaders, data analysts, financial analysts, and health care providers. We conducted 8 ARcare interviews with 12 participants and 8 Terros interviews with 9 participants.

We coded and analyzed transcripts in Dedoose version 9.0.46 (SocioCultural Research Consultants, LLC). Evaluation questions and constructs from the Consolidated Framework for Implementation Research (patient needs and resources, relative advantage, culture, implementation climate) guided codebook development (13). The codebook guided deductive coding of transcripts, and codes were added or revised inductively (14). To increase validity, all coders analyzed 1 transcript together. The remaining transcripts were independently coded and reconciled by 2 coders each, and they discussed disagreements until consensus was met. The following themes emerged during thematic analysis: telehealth use, community awareness of telehealth, patient barriers and facilitators to health care, telehealth as a barrier and facilitator to health care, and impact on blood pressure outcomes.

### Quantitative data sources and analysis

Each health care system exported de-identified data from their electronic health record and population health tools for before and after telehealth implementation due to COVID-19: March 1, 2019, through March 31, 2021, for ARcare, and March 1, 2019, through August 31, 2021, for Terros.

Data sets from ARcare and Terros had different variables and formats, but we used similar statistical methods. Study populations from both sites included patients with a diagnosis of hypertension, a primary care visit, and a blood pressure measurement during the observation period (ARcare, N = 574; Terros, N = 986). We calculated frequencies, percentages, and means (SDs) for patient demographic characteristics, patient clinical characteristics, and type of patient encounter (eg, telehealth, in-person). We defined blood pressure control as <140/90 mm Hg. We calculated blood pressure control rates for patients in the study population (number of patients with controlled blood pressure divided by the total number of patients with hypertension) for each site during the observation periods before and after telehealth implementation due to COVID-19. We used χ^2^ and Fisher exact tests to assess differences in patient encounter type across patients’ race and ethnicity and blood pressure control rate across observation periods.

We analyzed data in SAS version 9.4 (SAS Institute Inc) and Stata version 17 (StataCorp LLC) and visualized data by using Excel 2019 (Microsoft Corp).

## Results

Of 574 patients at ARcare, 57.3% were female, 64.5% were White, 34.1% were Black, and 1.0% were Hispanic; 35.7% received Medicare (Table). Of 986 patients at Terros, 43.7% were female, 71.6% were White, 18.9% were Black or African American, and 21.5% were Hispanic.

**Table:** Characteristics of Patients With Hypertension at ARcare and Terros Health^a^

Characteristic	ARcare (March 1, 2019–March 31, 2021)^a^	Terros Health (March 1, 2019–August 31, 2021)^a^
**Total no. of patients**	574 (100.0)	986 (100.0)
**Sex**		
Male	241 (42.0)	555 (56.3)
Female	329 (57.3)	431 (43.7)
Unknown	4 (0.7)	0
**Race**		
American Indian or Alaskan Native	0	14 (1.4)
Asian	1 (0.2)	16 (1.6)
Black or African American	196 (34.1)	186 (18.9)
Latino	—b	1 (0.1)
Native Hawaiian and Other Pacific Islander	1 (0.2)	7 (0.7)
White	370 (64.5)	706 (71.6)
More than 1 race reported	—b	4 (0.4)
Unknown or declined	6 (1.0)	52 (5.3)
**Ethnicity**		
Hispanic	6 (1.0)	212 (21.5)
Non-Hispanic	534 (93.0)	711 (72.1)
Unknown	34 (5.9)	63 (6.4)
**Insurance status**		
Medicaid	73 (12.7)	— b
Medicare	205 (35.7)	— b
Private	193 (33.6)	— b
Self-pay	93 (16.2)	— b
Other	10 (1.7)	— b
**Vitals at most recent visit, mean (SD)**		
Age, y	54.4 (13.6)	53.0 (12.1)
Systolic blood pressure, mm Hg	137.4 (19.3)	142.6 (20.5)^c^
Diastolic blood pressure, mm Hg	83.0 (11.1)^d^	70.9 (10.0)^e^
Pulse	— b	68.1 (28.5)^f^

a ARcare is a federally qualified health center (FQHC) that comprises 48 clinics that serve 17 counties in Arkansas, Kentucky, and Mississippi. Terros Health serves a diverse population at 13 locations in Arizona, including 4 FQHCs. Values are number (percentage) unless otherwise indicated.

b Data not collected.

c Data missing for 286 patients.

d Data missing for 2 patients.

e Data missing for 287 patients.

f Data missing for 313 patients.

### Reach to patients

#### Use of telehealth

Both sites had similar trends in telehealth use, before and after the transition to telehealth caused by COVID-19 (Figure). The proportion of telehealth encounters at ARcare was small (<10%) before the pandemic, and Terros had not yet implemented telehealth. Use of telehealth peaked at 65% (348 telehealth encounters of 537 total encounters) in April 2020 at ARcare and almost 90% in April (342 telehealth encounters of 384 total encounters) and May 2020 (284 telehealth encounters of 321 encounters) at Terros. After April 2020, the proportion of telehealth encounters for ARcare declined through March 2021 to 19% (70 telehealth encounters of 371 total encounters) but remained higher than before the pandemic. Similarly, for Terros, telehealth visits declined to 39% in August 2021 (119 telehealth encounters of 308 total encounters). The relationship between type of patient encounter and patients’ race (Terros *P* < .001) and race and ethnicity (ARcare *P* < .001) was significant at both sites (Appendix Supplemental Table 1 and Table 2). Although the study population at ARcare included few Hispanic patients (n = 6), Hispanic patients had the highest percentage of telehealth encounters (28%; 16 of 57) compared with other patients. Despite having small cell sizes for other patients, a high percentage of telehealth use was observed for White patients in Terros’ study population during their most recent encounter (44%; 314 of 706). Staff at Terros qualitatively expressed that the telehealth adoption rate for “everybody else, [was] slow and very steady and it remained disparate.”

**Figure:**
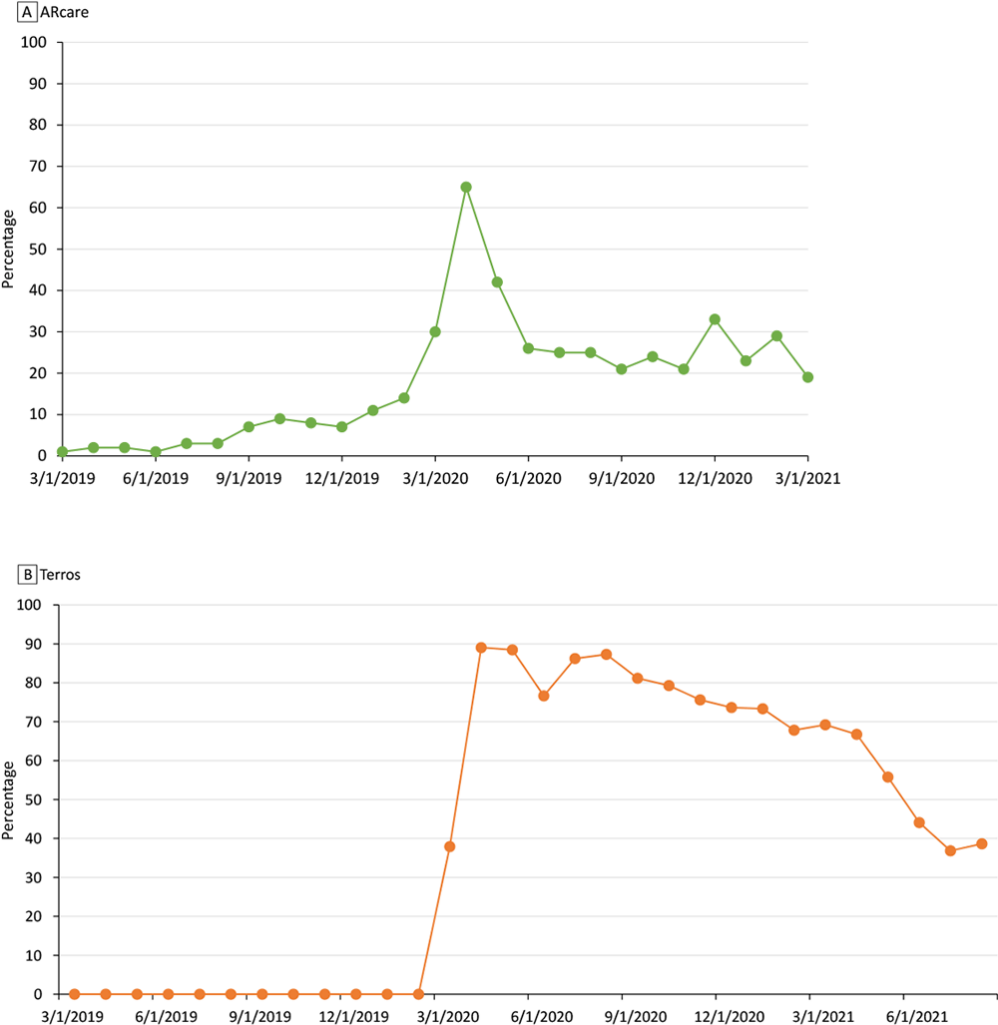
Percentage of encounters that were telehealth encounters among patients with a diagnosis of hypertension in A) ARcare and B) Terros health care systems, 2019–2021.

**Table:** 

A. ARcare				
Date	In-person, number	Telehealth, number	Total, number	Percentage of encounters that were telehealth encounters
3/1/2019	505	6	511	1
4/1/2019	501	10	511	2
5/1/2019	470	10	480	2
6/1/2019	393	4	397	1
7/1/2019	459	12	471	3
8/1/2019	521	14	535	3
9/1/2019	437	31	468	7
10/1/2019	544	52	596	9
11/1/2019	465	42	507	8
12/1/2019	840	59	899	7
1/1/2020	275	34	309	11
2/1/2020	267	44	311	14
3/1/2020	268	115	383	30
4/1/2020	189	348	537	65
5/1/2020	249	178	427	42
6/1/2020	329	118	447	26
7/1/2020	294	96	390	25
8/1/2020	281	95	376	25
9/1/2020	322	85	407	21
10/1/2020	335	107	442	24
11/1/2020	242	65	307	21
12/1/2020	227	113	340	33
1/1/2021	290	86	376	23
2/1/2021	183	76	259	29
3/1/2021	301	70	371	19
**B. Terros**				
3/1/2019	317	0	317	0
4/1/2019	261	0	261	0
5/1/2019	362	0	362	0
6/1/2019	312	0	312	0
7/1/2019	313	0	313	0
8/1/2019	349	0	349	0
9/1/2019	371	0	371	0
10/1/2019	403	0	403	0
11/1/2019	326	0	326	0
12/1/2019	284	0	284	0
1/1/2020	357	0	357	0
2/1/2020	380	0	380	0
3/1/2020	272	166	438	38
4/1/2020	42	342	384	89
5/1/2020	37	284	321	88
6/1/2020	88	289	377	77
7/1/2020	44	275	319	86
8/1/2020	43	295	338	87
9/1/2020	66	284	350	81
10/1/2020	69	264	333	79
11/1/2020	68	211	279	76
12/1/2020	83	232	315	74
1/1/2021	83	228	311	73
2/1/2021	111	234	345	68
3/1/2021	122	274	396	69
4/1/2021	116	233	349	67
5/1/2021	149	188	337	56
6/1/2021	223	176	399	44
7/1/2021	223	130	353	37
8/1/2021	189	119	308	39

ARcare and Terros staff members (5 ARcare interviews, 3 Terros interviews) indicated that convenience was perceived as a primary reason among patients for using telehealth and was often appreciated after an initial telehealth encounter. Both sites indicated that telehealth reduced appointment no-show rates, an idea that was conveyed by a health care provider at Terros who perceived that “e-visits are kept more by patients . . . because our patient population faces some transportation barriers, so for them to be able to connect to the e-visit, it seems to have been easier for them.”

#### Community awareness of telehealth

ARcare promoted community awareness of telehealth at satellite clinics via signs describing telehealth services and booths to disseminate information. Terros marketed telehealth services, informed patients about the convenience of same-day telehealth appointments, and staff, including community health workers, reached out to eligible patients, explained telehealth options, helped with connection issues, and worked with hesitant patients.

#### Patient barriers and facilitators to accessing health care

Social determinants of health that adversely affect patients’ overall access to health care (eg, lack of income, homelessness, lack of health care providers, language barriers) were described nearly universally (7 ARcare interviews, 7 Terros interviews). Transportation was commonly identified as a barrier to using traditional in-person care, and telehealth was viewed as improving access to care for patients experiencing some of these upstream barriers.

#### Telehealth as a facilitator and barrier to accessing health care

Each health system (4 ARcare interviews, 5 Terros interviews) offered insights into how telehealth facilitates access to care and can increase touchpoints within health systems. ARcare further noted the utility of telehealth for patients living in rural areas. Terros described the usefulness of telehealth for patients who cannot take time off work or lack childcare.

Conversely, both health systems described how telehealth can exacerbate a lack of health care access. Common patient barriers to engaging in telehealth were lack of access to equipment (eg, computer, smartphone, e-mail address, blood pressure monitor) and broadband internet. Other patients faced barriers because of limited data plans, poor internet connectivity, or gaps in technical knowledge and skills, which limited the information that could be gathered by health care providers and sometimes resulted in switching to telephone-only or an in-person visit.

### Blood pressure outcomes

For patients at ARcare, we found no significant differences in blood pressure control between baseline (53.4%, March 2019–February 2020) and after expansion of telehealth services (57.4%, March 2020–March 2021) (*P* = .31).

At Terros, 85.7% of patient visits were missing data on a systolic blood pressure reading, which was corroborated during interviews. Staff indicated that few patients who engaged in telehealth had a blood pressure monitor, but these patients were perceived as “more adherent and consistent” because of telehealth.

## Implications for Public Health

These rapid evaluations explored how telehealth strategies delivered during the COVID-19 pandemic in a primary care setting affected use of telehealth, potential disparities in use by race and ethnicity, and blood pressure outcomes in 2 health care systems. Both systems had similar trends in telehealth use that align with the literature and federal reports (8–10). A review of telehealth studies conducted during the COVID-19 pandemic supports our finding of conflicting evidence on telehealth use and patient race and ethnicity (10). ARcare and Terros promoted community awareness of telehealth, and health systems could consider implementing and evaluating additional approaches that address upstream barriers to use.

ARcare’s blood pressure control rates did not decrease after telehealth implementation, which supports the adequacy of telehealth for primary care services. ARcare’s clinic-to-clinic telehealth approach of outfitting local clinics with technology might lessen patients’ technology and transportation barriers while allowing for consistent blood pressure measurement. A similar telehealth approach at Veterans Affairs hospitals demonstrated parity with in-person diabetes care (15). A nationally representative study of outpatient care in the US during the COVID-19 pandemic (7) helps explain why changes in blood pressure control could not be calculated for patients at Terros. The study found that blood pressure assessment was significantly less common among telemedicine encounters compared with in-person (7). Emerging evidence suggests the need for interventions that support consistent data collection during telehealth encounters.

Qualitative findings from ARcare and Terros suggest that telehealth offered consistent or improved health care for some patients with a diagnosis of hypertension during the COVID-19 pandemic but not for patients lacking technology or internet, suggesting that telehealth is not a universal solution and requires tailoring to some populations. Similar barriers to health care and engagement in telehealth have been reported (8,10,11). Relaxed regulations during the COVID-19 pandemic allowed health systems to deliver audio-only telehealth and improve opportunities for use among patients lacking technology or broadband internet (11). Long-term sustainability of these policy changes is uncertain, but national authorities recommend extending flexibilities and prioritizing broadband internet as a social determinant of health (11). Policy levers may foster equitable access to health care services that support hypertension management and control.

This rapid evaluation has limitations. Findings are not generalizable to all US health care systems but may expand the evidence base. We were unable to interview patients, but staff reported patient barriers and facilitators to accessing health care and telehealth that align with published literature (10,11). Quantitative findings should be interpreted cautiously because study population sizes were small and were not adjusted for confounders or bias. We could not calculate blood pressure control for Terros, but this illuminated a barrier to improving the use of telehealth for hypertension management and control. Practice-based evidence generated by these rapid evaluations suggest that telehealth offers consistent health care access and blood pressure outcomes for some patients with hypertension, but health inequities may be exacerbated among patients with barriers to telehealth and blood pressure measurement.

## Appendix Supplemental Tables

**Appendix:** Supplemental Table 1. Telehealth Encounters Among 574 Patients, by Race and Ethnicity, ARcare, March 1, 2019–March 31, 2021

Race and ethnicity	No. of encounters (% of encounters that were telehealth)^a^	*P* value^b^
Non-Hispanic White	1,152 (15.8)	<.001
Non-Hispanic Black	687 (18.8)	
Hispanic	16 (28.1)	
Other	15 (25.0)	

^a^ Determined by χ^2^ test and compares uptake of patient encounter across patients’ race and ethnicity.

^b^ Patients could have had ≥1 telehealth encounter.

**Appendix:** Supplemental Table 2. Telehealth Use by Modality (Videoconferencing and Telephone-Only) and Overall Among 986 Patients, by Race and Ethnicity, Terros Health, March 1, 2019–August 31, 2021

Characteristic	No. of videoconference visits (% of all visits)	No. of telephone-only visits (% of all visits)	Total no. of telehealth visits (% of all visits)	*P* value^a^
**Race**				
American Indian or Alaska Native	1 (7.1)	5 (35.7)	6 (42.8)	<.001
Asian	1 (6.3)	7 (43.8)	8 (50.1)	
Black	22 (11.8)	55 (29.6)	77 (41.4)	
Latino	1 (100)	0	1 (100)	
Native Hawaiian or Other Pacific Islander	0 (0)	2 (28.6)	2 (28.6)	
White	90 (12.8)	224 (31.7)	314 (44.5)	
More than 1 race	2 (50.0)	0	2 (50.0)	
Unreported	6 (11.5)	14 (26.9)	20 (38.4)	
**Ethnicity** ^b^				
Hispanic	18 (8.5)	77 (36.3)	95 (44.8)	.13
Not Hispanic	95 (13.4)	215 (30.2)	310 (43.6)	
Unknown	10 (15.9)	15 (23.8)	25 (39.7)	

^a^ Determined by Fisher exact test because of small cell size; compares uptake of patient encounter (ie, videoconferencing, telephone-only, in-person) across patients’ race and ethnicity, respectively. Data on in-person encounters are not presented.

^b^ Total number of telehealth encounters differs between race and ethnicity because of missing information on ethnicity.
